# Anti-racist learning in the university: trilemmatic positionality in teaching racialization processes in Germany

**DOI:** 10.3389/fsoc.2025.1584741

**Published:** 2025-12-04

**Authors:** Maisha M. Auma, Gökce Yurdakul

**Affiliations:** 1 Universitätszentrum für Frauen- und Geschlechterstudien (UZF^*^G), University of Klagenfurt, Klagenfurt, Austria; 2 Institut für Sozialwissenschaften, Berlin Institut für Migrations- und Integrationsforschung (BIM), Humboldt-Universität zu Berlin, Berlin, Germany

**Keywords:** critical race pedagogy, trilemmatic positionality, higher education, racialization, Germany, anti-racist learning

## Abstract

This article analyzes the pedagogical and institutional challenges of teaching about racialization processes in Germany through a BA-level seminar, Processes of Racialization in the “Two Germanies. ” Drawing on critical race pedagogy and the concept of trilemmatic positionality, we explore how students navigated tensions between institutional resistance, anti-racist commitments, and their own lived experiences. Based on students' reflections and feedback, the article examines how language, digital learning, and institutional structures shape anti-racist learning. Our findings reveal the emotional labor of students, the discomfort of privileged learners, and the limits of institutional recognition of racism. We argue for the institutionalization of anti-racist pedagogy in German higher education and highlight how trilemmatic positionality provides an analytical tool for understanding the contradictions and potential of anti-racist teaching.

## 1 Introduction

This article examines the challenges and opportunities of teaching about racialization in Germany. We draw on our BA-level seminar Processes of Racialization in the “Two Germanies” as a case study to reflect on how students engaged with anti-racist learning in a resistant institutional context. The concept of trilemmatic positionality, the tension between institutional constraints, anti-racist commitments, and lived experience, serves as our analytical framework. Despite Germany's democratic and humanist educational ideals, the legacy of Humboldtian universalism has often marginalized discussions of race and racism. Within this landscape, our seminar sought to foreground structural racism and belonging across both German states. We asked students to reflect on their own learning processes through this trilemmatic positionality in a political environment in Germany that resists public discussion of racial inequalities and critiques of racism.

In Germany, education often framed social inequality through class or ideology, while sidelining structural racism and colonial legacies. Specifically in German higher education, teaching race and racism remains institutionally marginalized and pedagogically fraught. Our seminar was unique in several ways for German universities. First, it was conducted in English, marking a departure from a predominantly German-language curriculum and mode of instruction. This decision attracted an extremely diverse group of students that included both white and white, migrantized and minoritized German students and international students. Second, we adopted an interdisciplinary approach, drawing on sociology, history, and critical race studies to examine racialization processes in East and West Germany. Third, the seminar examined the “two Germanies” rather than privileging West Germany, as is common in most German academic discourses on race and racism. Last but not least, we offered the seminar online in order to accommodate our schedules in different countries as well as to offer students a smoother transition to in-person learning after COVID-19. Beyond these pedagogical choices, we are also unusual scholars in German academia: We are first-generation migrants to Germany—one from Kenya and the other from Turkey—and are German nationals. Our perspectives informed the course's critical approach to racialization and its emphasis on diverse forms of belonging and experiences of racism. We assume that our identities also affected how students related to the seminar and to our discussions.

This article takes the seminar as a case study to examine the challenges and opportunities of teaching about racialization in contemporary Germany. Drawing on student discussions, short written reflections, and anonymous course evaluations, we explore how students navigated tension between the three simultaneous and often conflicting pressures of trilemmatic positionality: (1) the institutional constraints and silences around teaching about racism in German academia; (2) students' personal commitments to anti-racist learning and social justice; and (3) students' own lived experiences and positionalities in relation to race, migration, and power. Rather than a linear or resolved engagement, students moved in and out of these positions throughout the seminar, sometimes embracing discomfort and self-reflection, and at other times resisting or withdrawing when confronted with challenging material. This trilemma shaped their engagement with course content and each other, revealing both the pedagogical challenges and the transformative potential of teaching race and racism in a context historically resistant to discussing it.

This article is structured as follows: We first situate our teaching approach within the broader landscape of post-unification Germany, focusing on the resistance to racism research and critical race pedagogy at German universities. We then contextualize our perspective within current debates on anti-racist teaching in Germany. Following this, we present the course design and reflect on its implementation. Our findings highlight the different ways students engaged with themes, such as emotional and ethical reflections, and the role digital spaces and language barriers play in teaching about race and racism. We conclude by discussing the broader implications for anti-racist pedagogy in German higher education, emphasizing the importance of creating spaces that acknowledge both historical complexities and institutional constraints.

## 2 Resistance to racism research and critique in the GDR, FRG, and post-unification Germany

Only after the intertwined crises of the COVID-19 pandemic and the global wave of anti-racism movements in the wake of 2020 did serious attention begin to turn, within German academia, to the country's historical and ongoing experiences of racism (Alexopoulou, 2021; Grau, 2022; Auma, 2020). This shift marks a departure from earlier reluctance to engage with race as a category of analysis in Germany, where discussions of racism have often been overshadowed by the country's reckoning with its antisemitic and colonial past (El-Tayeb, 2016; Gelbin et al., 1999). However, this newfound openness to considering racism beyond the Third Reich's legacy remains fraught with challenges, and the history of how race became an analytical category during the racialization processes in both East and West Germany remains especially difficult.

In this context, we define race as an analytical but constructed category based on socially significant characteristics. As sociologists Murji and Solomos (2005) show, race is not just a neutral label, but a socially constructed idea that has been used throughout history to exclude and disadvantage certain groups, both through broad explanations for inequality and through specific cultural or political systems that reinforce those divisions. We emphasize that race is not a biological reality, but rather a social construct that has been used to categorize and often hierarchize groups of people. As race is constructed, our analysis turns to the racialization processes, by which we mean how the concept of race comes to be applied to individuals, groups, or social practices. This process involves the attribution of racial meanings to people's identities, relationships, and social structures that were previously unclassified in racial terms. In our understanding of racialization, we join art and cultural historian Lewis (2024, p. 6), who aptly stated that “racial observation is not just about what is seen, but also about what can be disregarded and how”. Race becomes embedded in how we interpret social life, not only through recognition, but through strategic misrecognition or erasure (for example in the case of erasing racialization processes of the GDR). This analytical understanding of race allows us to define racism as a systemic issue, deeply rooted in colonial, social, and institutional structures, a political system of oppression and exploitation (Mills, 1997, also see Erel et al., 2016).

The hegemony of West German perspectives defining race and racism in the German context requires rigorous interrogation. A major challenge in teaching about race in Germany is confronting the hegemony of West German perspectives in shaping both academic and public discourse. The FRG's racialization dynamics, particularly in relation to labor migration, have dominated discussions of race and racism in Germany, often sidelining the GDR's specific racialized histories. This imbalance reinforces an implicit West German normativity, where East German experiences of racialization are either erased or framed as exceptions to an assumed “Western” model.

Two definitions of key concepts are needed before we go further: The first is Rassisifizierung, a German term which we translate as processes of racialization that refers to the process by which individuals, groups, practices, or spaces are ascribed racial meanings, often in ways that naturalize difference and justify racial inequality. Racialization is not only about overt hostility but includes subtle, structural, and symbolic processes that produce and reproduce racial categories and hierarchies. Ulimately, racialization reflects how racism operates beyond individual prejudice, shaping institutions and knowledge systems. In the German context, the concepts of Rasse and Rassifizierung have a long and fraught history, deeply shaped by colonial projects, racial science, and the catastrophic racial ideology of National Socialism. After 1945, explicit references to Rasse were delegitimized in public discourse, yet processes of Rassifizierung continue to structure social hierarchies and exclusions in social institutions through law, migration regimes, policing, and everyday discrimination.

The second key concept is race project. Drawing on Kimberlé Crenshaw's intersectional legal critique, which is central to Critical Race Theory, the idea highlights how anti-discrimination laws are never neutral or universally applicable. Instead, they are shaped by each nation-state's particular historical, legal, and political relationship to racism or its race project. From this perspective, anti-discrimination law does not express an abstract or universal commitment to equality, but rather the limits of what each legal system is able (or willing) to recognize as racial harm. For example, in the GDR (East Germany), where the state defined itself as fundamentally anti-fascist, racism was officially denied altogether. By contrast, in the FRG (West Germany), racism was acknowledged, but treated as a marginal or exceptional problem rather than a structuring force of society. Crenshaw's formulation of “specific race projects” helps us see how national histories and ideologies shape different forms of exclusion—and with them, different limits to legal protection. Her more recent work with Critical Race Theory Europe, and her advocacy for a European CRT network, underscores why this framework is valuable for analyzing racial formations and their legal consequences in a European context (Auma, 2019; Crenshaw, 1991).

Turning back to the racialization processes in the two Germanies, we find distinct, yet interconnected, race projects in each German state. While officially promoting anti-fascism and socialist internationalism, the GDR developed a racialized labor system that positioned migrant workers from socialist “brother states” within a rigid hierarchy. The Vertragsarbeit (contractual work) system exemplified this contradiction: Workers were framed as guests who contributed to socialist solidarity, yet they were structurally marginalized and excluded from long-term social integration. Meanwhile, the FRG's economic expansion was underpinned by Arbeitsmigration (workers migration), which recruited labor from Southern Europe, North Africa, Turkey, and beyond. Despite an official discourse of “integration,” labor policies reinforced racial hierarchies, rendering many migrants structurally precarious (Herbert, 1990).

The GDR's race project was highly contradictory and its anti-racism paradigm deeply ambivalent. On the one hand, the state supported anti-colonial movements and framed itself as an ally to newly independent nations. On the other hand, this rhetorical commitment to international solidarity often masked deeply racialized labor structures within the country. Programs like Vertragsarbeit allowed the state to recruit migrant labor while maintaining strict limitations on residency, rights, and integration. Workers from socialist “brother states” were simultaneously celebrated as symbols of global solidarity and confined to precarious conditions that formed the hard limits of socialist egalitarianism. Teaching these contradictions required an approach that could interrogate the gap between ideology and lived realities—a crucial methodological challenge when addressing racialization in either Germany. The FRG's migration regime was also shaped by a fundamental ambivalence: while West Germany projected itself as a liberal democracy open to international cooperation, its Gastarbeiter system was firmly rooted in the logic of temporary labor migration and a persistent refusal to recognize itself as a country of immigration. Turkish and other migrant workers were recruited to fill labor shortages, yet their presence was framed as provisional, with policies such as the Rotationsprinzip (rotation principle) reflecting a clear expectation of migrant workers' eventual return rather than their integration (Yurdakul, 2009). This approach marginalized migrants by restricting their rights to residency, family reunification, and citizenship, relegating them to precarious, low-status jobs and systematically excluding them from full participation in West German society. The murders of migrants and their families, such as the Arslan and Yilmaz families in Mölln and the Genç, Öztürk, and Ince families in Solingen, exposed the deadly consequences of a society that persistently framed migration as a problem and migrants as culturally incompatible to Germany's Leitkultur (dominant culture). West Germany—and reunified Germany, which as we will discuss cannot be simply equated with the FRG—has lived in a conundrum between economic dependence on migrant labor and the denial of their belonging in German society. Processes of racialization, migration, and Germany's national identity remain central to contemporary debates on belonging and citizenship (Korteweg and Yurdakul, 2024).

While scholarship on racism in Germany has grown significantly, it remains dominated by a West German lens (Räthzel, 2012) and thus erases the inheritance of East German racial relations (see Lewis, 2024, on erasure). To treat Germany as simply West Germany overlooks the unique historical and political-economic conditions of the GDR, where racialization operated under the guise of socialist internationalism (Piesche, 2016). Especially the post-unification period of the 1990s, marked by extreme racist violence and hate crimes (often referred to as the Baseballschläger-Jahre^1^), is frequently discussed without sufficient attention to the continuities and ruptures between East and West German histories of racism (Waibel, 2016; Warda, 2022). This article addresses these gaps by examining processes of racialization in both German states, from the post-World War II era to the present, and by reflecting on the methodological challenges of teaching this history and present in an academic setting.

In addition to the questioning of a West-German-centric perspective on racism, recently German academia has been confronted by decolonial perspectives that center voices historically sidelined in dominant discourses of race and that challenge the Eurocentric assumptions embedded in German sociology and race studies (El-Tayeb, 2016; Auma, 2020). Critical race theory provided a foundation for interrogating the power relations inherent in racialization processes, allowing us to analyze how racism operates structurally and to critique the ways in which academic knowledge production reproduces racial hierarchies (Gelbin et al., 1999).

An important strand of racism research in Germany after the unification focuses on anti-Muslim racism and seeks to highlights the skewed portrayal of Muslims in public discourse and policy (Özvatan et al., 2023). Research shows that Muslims in Germany are often framed as subjects of integrationist policies, a framing that reinforces stereotypes of passivity and otherness. As Rothberg and Yildiz (2011) argue, these narratives are structurally restrictive and ethnically dominant, positioning Muslims as passive recipients of German history and national narratives. Contrary to their portrayal in debates on Leitkultur, state neutrality, and gender equality, young Muslims in Germany are actively producing alternative narratives that challenge stereotypes and assert their belonging in German society. These narratives, often disseminated through the internet and independent publications, represent a powerful counterpoint to mainstream media portrayals. Initiatives like the German Islam Conference and the Young Islam Conference have played a significant role in fostering these alternative narratives, creating spaces for dialogue, and redefining what it means to be German in contemporary society. However, these efforts remain constrained by broader structural and institutional barriers, reflecting the ongoing marginalization of anti-Muslim racism research within German academia.

The racism research in post-unification Germany has merged with discussions about the racialization of immigrants, mostly Turkish immigrants, which have been increasingly perceived as Muslims (Yurdakul, 2009). The continuous violent attacks toward immigrants in Germany, including the arson attacks in Solingen (1993) and Mölln (1992), the xenophobic riots in Rostock-Lichtenhagen (1992) shortly after reunification, followed by the series of murders committed by the National Socialist Underground (NSU) between 2000 and 2007, and more recently the terrorist attacks in Hanau (2020) demonstrate a persistent pattern of right-wing extremist violence and racism that has plagued the country in the post-unification period. But to discuss these attacks only in the terms of migration research can fail to address the central place of racism itself. For example, the concept of postmigration has gained traction in German academia as a framework for analyzing how societies transform through migration, and this framework situates racism as a structural problem in postmigration societies. Scholars have used this framework to explore migrants' experiences, historical legacies, and colonial and postcolonial conditions (Foroutan, 2019; Römhild, 2021; Römhild et al., 2018). While postmigration offers valuable insights into societal transformations, one of its major challenges is the persistent migrantization of racialized people, often implying that racialized people are inherently considered as non-belonging to their nation-states (Korteweg and Yurdakul, 2024)^2^.

While racism research in Germany faces institutional challenges, there has been significant scholarly attention and research on right-wing political parties, particularly the Alternative for Germany (AfD). Researchers have analyzed the AfD's electoral success, its support base, and the factors contributing to its growth (Manow and Schwander, 2022; Hansen and Olsen, 2022). Studies have also examined the party's rhetoric, policy positions, and links to far-right movements (Doerr, 2021). These analyses provide valuable insights into the broader context of right-wing populism and its impact on German politics and society. The disproportionate focus on the AfD and right-wing populism in discussions about racism in Germany often overshadows the deeper, more pervasive issues of structural and institutional racism, a tendency to view racism as an isolated problem confined to far-right groups. This narrow focus reflects the broader institutional resistance to addressing racism in a comprehensive and intersectional manner that concerns German society as a whole.

## 3 The challenges of teaching about racialization processes in Germany

In this socio-political context, there are three challenges in teaching about racialization processes in the “two Germanies” at a German university:

Fragmented literature: The first challenge is to decide the content and scope of literature we can include in the course readings. The sociological literature and data on racism is fragmented. This fragmentation stems from the erasure of racism and critiques of racism in the GDR and other post-socialist societies in Eastern Europe. While West Germany's history of racism has been more extensively documented, the GDR's official narrative of socialist internationalism and anti-fascism often obscured the realities of racialization within its borders (Räthzel, 2012; Piesche, 2016; Pilz, 2020). As a result, to piece together a coherent understanding of race, racialization processes, and racism in both German states, scholars and educators must navigate a patchwork of sources. This fragmentation is also reflected at the level of data collection: the divided history has created significant barriers to comprehensive demographic data collection on racism in Germany.

The marginalization of racism research has also been compounded by a postwar reluctance in either Germany to collect demographic data on ethnicity. This reluctance stems from the troubling legacy of the Nazi regime, which used data collection and census programs to target and persecute Jews, Romani people, and other minorities. The Nazis' exploitation of demographic data to facilitate mass murder has left a deep-seated wariness of data gathering efforts, even when the intent is benign. Without robust demographic data, contemporary scholars struggle to substantiate their claims, often leading to their work being dismissed as anecdotal or lacking empirical support. In the German social sciences, where many colleagues rightfully take pride in long-established and well-funded statistical databases, research on racism that lacks comparable statistical evidence has been viewed as a topic that lacks rigorous scientific backing. However, recent efforts, such as the Afrozensus (2020), (https://afrozensus.de) and the Rassismusmonitor (2023), (https://www.rassismusmonitor.de/), have sought to address this gap by collecting statistical data on racism.

Beyond the projects of collecting the evidence necessary to make quantitative arguments about race and racism in Germany, scholars have also focused on developing qualitative research on race and racialization. Two examples to vast amount of qualitative research (see Melter and Mecheril, 2009 for an overview) include Auma's project “Making Visible the Discrimination and Social Resilience of People of African Heritage in Berlin” (Auma, 2018), in which Auma conducted interviews and focus groups with racialized people in Berlin to document their everyday experiences with racism and their strategies of empowerment and resistance. Beyond recording discrimination, the project sought to make visible the resources and resilience of the participants in order to develop concrete, actionable recommendations for policy and society. Another example is Yurdakul and Altay's research on how Turkish immigrant mothers create strategies to overcome migrant stigma in Germany's educational institutions (Yurdakul and Altay, 2023). If both qualitative and quantitative research in Germany aim to make racism visible as a social issue and to provide an evidence base for developing effective anti-racism discourses and policies, in the absence of comprehensive quantitative data, qualitative research methods, such as ethnographic studies, interviews, and narrative analyses, have become vital tools for understanding the experiences of racialized communities in Germany, centering the voices and experiences of those directly affected by racism.

Contested terminology: The second challenge of teaching about race in Germany is the controversy surrounding the terminology used to discuss racism. Haunted by its antisemitic and racist past, Germany has struggled to find a language that adequately addresses contemporary racism without evoking the language and atrocities of the Third Reich. As scholars like Yurdakul and Altay (2023) and Arnold (2018) have noted, this linguistic ambivalence reflects broader societal discomfort with confronting racism as a present-day issue. Despite this, seminal works such as After the Nazi Racial State (Chin et al., 2009) have demonstrated that race did not vanish from public discourse or policy-making after the defeat of the Third Reich. Instead, race continued to shape societal attitudes and policies in both Germanies and across Europe, albeit in different ways.

Institutional marginalization: The third challenge we encountered is Germany's institutional resistance to talking about racism. The study of racism in German academia remains a contentious issue, shaped by historical, political, and institutional factors that undermine the legitimacy of racism as a scholarly topic. In our scholarly discussions, we witnessed that some scholars contend that overt racial ideologies belong exclusively to the Nazi regime and are no longer prevalent in contemporary Germany. This perspective overlooks the enduring impact of historical racism and the ways in which racial discrimination persists in contemporary Germany. By insisting that racism is a relic of the past, this argument obscures the structural and systemic nature of racial inequality today. Another form of institutional resistance appears in the argument that racism is a left-wing activist topic and lacks the scholarly rigor required for academic inquiry. This claim reflects a misguided perspective that fails to recognize the pervasive nature of racism within educational institutions and the broader society. Addressing racism through scholarly research and teaching is not only academically valid but also essential for understanding and dismantling systemic inequalities.

Despite these challenges, significant strides have been made in recent years to institutionalize racism research in Germany. A key development in this regard is the establishment of the National Discrimination and Racism Monitor (NaDiRa) by the German Center for Integration and Migration Research (DeZIM). Initiated in response to a mandate from the German Bundestag in July 2020, NaDiRa was funded to develop a comprehensive monitoring system for racism and discrimination in Germany. Building on existing research and activist knowledge, NaDiRa has made racism a salonfähig (socially acceptable) topic in academic and public discourse. NaDiRa's systematic studies have shed light on the causes, extent, and consequences of racism in Germany, providing a robust evidence base for anti-racism policies and interventions. However, the institutionalization of racism research remains limited, with funding and recognition often lagging behind other areas of study. This lack of support reflects broader societal ambivalence about confronting racism and underscores the need for continued advocacy and investment in the field.

Today, racism research in Germany remains an underdeveloped and poorly funded field, factors which effectively marginalize it in the broader academic landscape. Unlike more established disciplines, there are very few dedicated research institutions or professorships specifically focused on racism studies (e.g., one exception is Audre-Lorde Professorship at the Technical University of Berlin, there are a few such professorships around Germany). This lack of institutional support limits the visibility and impact of the field, forcing scholars to conduct research within adjacent disciplines such as migration studies, diversity studies, and gender studies. While these fields provide valuable interdisciplinary perspectives, they often fail to address racism as a standalone and central issue. For example, the Audre Lorde Professorship, funded by the Berlin University Alliance, represents a rare but limited-term initiative to support research and teaching on racism and anti-racism. Scholars like Maisha Auma and others have contributed significantly to this field through this platform, but the temporary nature of such positions underscores the precarious status of racism research in Germany. Efforts to institutionalize racism research, such as those by Maisha Auma, have also faced challenges due to limited funding and support. This reliance on short-term funding not only hampers the development of a comprehensive understanding of racism, it also marginalizes scholars working in this field, leaving their contributions undervalued and underrecognized. These silences and ambivalences create the institutional terrain in which anti-racist teaching must operate.

## 4 Methods: content, course design and pedagogical methodology

In preparing and teaching this course, we also wanted to document our teaching and the students' learning experience to make a contribution to critical race pedagogy in Germany and beyond. While a broader range of materials was collected throughout the seminar, such as students' reading reflections and final essays, our analysis in this article draws specifically on three resources: A digital notepad that we used to summarize the lectures, students' brief reflective exercises, and students' feedback forms. These sources are most relevant for this article's research question: How can students be equipped with the analytical tools for self-reflexivity and positionality?

To answer this question, we followed a standardized analytical procedure. First, we informed students verbally that their reflections might be used for the purposes of this article and obtained their verbal consent. We clarified that their withholding consent would have no impact on their participation or performance in the seminar. Second, we collected brief reflective essays from students over the course of the semester, and invited them to submit feedback forms at its conclusion. We analyzed this material using both inductive and deductive coding, which allowed us to develop the thematic categories presented in this article. Drawing on these reflections, we also critically engaged with the assigned readings and our own teaching methods during the writing process. Finally, we engaged in self-reflection on our co-teaching and co-authoring processes as two women scholars with migrantized and racialized identities, discussing how our identities might have affected the students' reflections.

We draw on Burawoy's (1998) Extended Case Method (ECM) as a foundational approach in this research. ECM begins with in-depth empirical observations of specific social contexts, often through ethnographic engagement, rather than starting from abstract theory. We began by applying ECM to our pedagogical methods. By grounding our analysis in this concrete case, ECM allows us to refine, extend, and critically challenge existing pedagogical and sociological theories. Highlighting how everyday experiences are embedded in larger institutional and historical processes, ECM also provides the theoretical linkage between the micro-level interactions we witnessed and took part in in the seminar and Germany's broader social structures. Most importantly, ECM demands reflexivity, and prompted us as researchers to critically consider our own positionalities and their impact on knowledge production. Through this approach, we aim not merely to illustrate familiar theoretical concepts, but to transform and deepen our understanding by situating students' engagement with race and racism in our seminar within the wider context of German universities' approach to teaching about racialization processes.

### 4.1 Content and course design

Our course was structured around four thematic blocks, each designed to provide students with a critical framework for analyzing racialization processes in the “two Germanies” and their enduring legacies. First, we introduced key concepts such as racism, racialization, and marginalization, drawing on interdisciplinary literature to provide a theoretical foundation. Second, we examined significant socio-historical events in both German states, tracing the evolution of racialization practices over time. Third, we focused on the GDR System, exploring the GDR's political economy which was officially oriented toward socialist internationalism but also perpetuated racialized labor policies through programs like Vertragsarbeit (contract work). Migrant workers from African, Latin American, and Asian nations, many of which had recently gained independence from colonial rule, were recruited to the GDR under labor policies that masked racial hierarchies beneath a rhetoric of solidarity. Fourth, we turned to the FRG, whose political economy, in contrast to the GDR's, was rooted in free-market capitalism and officially promoted integration. However, beneath this surface, racialized labor markets and marginalization practices persisted, particularly in the context of Arbeitsmigration (labor migration). This course block also examined post-unification racist violence and institutional responses in both East and West Germany, highlighting the critiques of racism that emerged from civil society and policy spheres. We concluded this section by discussing the racist violence against immigrants following the guest workers recruitment in the FRG through to the current postmigration debates.

The course also included two guest lectures. Two sociologists of color, Çagri Kahveci (socialized in Turkey and then in West Germany) and Katharina Warda (socialized in East Germany), delivered online-guest lectures based on their own writings about racialization in West and East German societies respectively (Kahveci, 2023; Warda and Poutrus, 2022). Warda's project “Dunkeldeutschland” (Dark Germany) explored the post-reunification period from the social margins, illuminating blind spots in German historiography. Warda's work is based on her own intersectional experiences as a Black East German woman in the GDR and after 1989/90. Kahveci is one of the editors and contributors to the book Solingen, 30 Jahre nach dem Brandanschlag (Solingen, 30 Years After the Arson Attack, 2023), which examines the racist arson attack that occurred in Solingen, Germany on May 29, 1993 that killed five members of a Turkish-German family (Demirtas et al., 2023). During his presentation, Kahveci gave concrete examples of anti-migrant racism, including perspectives from survivors, family members, and other victims of racist and far-right violence (Kahveci, 2023). Both Warda and Kahveci's perspectives enriched the course by providing embedded insights into racialization processes in pre- and post-unification German contexts.

### 4.2 Pedagogical concept

Our course was designed as a virtual learning environment, which enabled our students to participate from spaces of their own choosing, often from their own homes or in different countries of residence (one student, for example, was on an exchange semester in Israel; another student was traveling in different countries of the African continent). Compared to traditional university seminar rooms, this format created a less formal dynamic and fostered a sense of flexibility, fluidity and accessibility. Each session was recorded and accompanied by shared notes, ensuring course resources were highly accessible and allowed students to revisit discussions at their own pace. We conducted the course in English, which leveled the playing field for students whose first language was not German.^3^ This approach allowed many students to engage more confidently and draw on their competencies without the added pressure of navigating German-language barriers. At the same time, teaching in English within a predominantly German-speaking institution posed distinct challenges, such as the need for spontaneous translation of German-language materials and the risk of delegating discussions of race and racism to contexts outside Germany. The virtual format also facilitated a high level of student engagement, with critical thinking skills honed through more focused and intense discussions and break-out group sessions.

Our pedagogical concept further focused on student-centered forms of learning that empowered students to take more control of their learning experience. We achieved this by designating two student-curated sessions. The participants collectively agreed on two additional topics not originally included in the course design: “The Pogrom in Rostock-Lichtenhagen in 1992 and its Afterlife” and “An Introduction to Said's (1979) Seminal Work on Orientalism”.^4^ These sessions gave students space to explore areas of particular interest and relevance to them, thus deepening their engagement with the course material in general.

Another key aspect of our pedagogical approach was continuous collaborative documentation and resource sharing. Each week the lecturers exchanged their roles, one acting as a facilitator and taking notes on a digital notepad while the other moderated discussions, a system that ensured comprehensive and accurate documentation. This seminar-pad, a set of notes which formed the basis for this article, functioned as a living record of each session, enhancing the course's accessibility and service orientation. In addition, every week the lecturer's presentation was recorded and uploaded on the intra-university digital platform to allow students to catch up with the course material in the case that they could not attend. These recordings also became resources to write this article. Due to ethical considerations, students' discussions were not digitally recorded. However, students could contribute to the seminar notepad anonymously, fostering open and candid participation. Beyond summarizing discussions, the notepad compiled contemporary debates, related topics, podcasts, and blog articles, creating an extensive and participatory archive. Combined with the recorded sessions, our approach provided students with continuous access to course materials, allowing for deeper engagement with the content.

A critical dimension of our pedagogical practice was its intersectional and positionality-conscious approach. As educators of color and intersectional feminist scholars, we brought our own lived experiences of structural and everyday racism into the classroom. Drawing on Mai-Anh Boger and Nina Simon's “together-apart-collective learning” (zusammen-getrennt-gemeinsam) framework (Boger and Simon, 2016), we structured discussions to address the complexities of racialized and postmigrant subjectivities in a hyperdiverse classroom. This involved an iterative process of collective theoretical grounding, segregated group work (where white students critically examined white privilege, and students of color reflected on self-alienation and self-determined agency), and collective reconvening to strategize pathways forward.

This pedagogical design enabled students to connect their biographies with Germany's history and thereby deepen their understanding of racialization processes, as well as encouraging reflection on their subjective positions within broader structures of racialization in the two Germanies. Students explored experiences such as growing up in GDR-era families, navigating racism in Germany, and questioning racialization as white (West-) or East German individuals. In their final papers and seminar discussions, students critically engaged with the course material through these personal lenses. The students often linked their individual and family histories to Germany's socio-political context, demonstrating the course's transformative and unique contribution to make sense of their own lived experiences. This design allowed students to navigate the tensions of trilemmatic positionality across language, digital format, and institutional norms.

## 5 Findings: trilemmatic positionality and students' reflections

While fostering critical engagement with the course material, we incorporated structured feedback mechanisms to assess the seminar's impact and refine our pedagogical approach. A key moment of reflection was an intensive self-study session conducted at the three-quarter mark of the course, during which students were prompted to document their learning experiences and positionalities. This exercise required them to write a short essay responding to a set of guiding themes, specifically on the tensions of trilemmatic positionality (Boger, 2017; Boger and Simon, 2016).

Trilemmatic positionality describes the complex situation many students find themselves in when trying to do anti-racist learning in universities. It involves balancing three difficult roles at the same time. First, students are part of institutions, like universities, that may not fully support or may even resist anti-racist learning. Second, they are committed to creating change and challenging racism through their learning processes. Third, they are personally affected by issues of race and power, either because they experience racism themselves or because they hold some form of privilege. This creates a difficult balancing act: how can someone question the system while still learning? How can they stay practicing anti-racism in a university system, which does not engage with anti-racist education? Trilemmatic positionality helps us understand the emotional and ethical challenges that come with doing this kind of learning process.

We asked students to discuss their own situatedness within processes of racialization and encouraged them to discuss trilemmatic positionality with a choice of four prompts: addressing key challenges in studying racism in Germany: using English as the language of instruction, navigating the virtual learning environment (Zoom), grappling with racialization either from a marginalized or privileged position, and comparing the study of racism in Germany with experiences from other national or regional contexts. These alternative reflections provided additional insights into the complexities of student engagement with race and racism in an academic setting.

Institutional Norms and the Lack of Anti-Racism in Broader Curricula:

“I just wish studying (anti)racism was more common in other courses, I for example studied law at HU before, an inherently very white faculty, and the curriculum didn't involve any lectures or seminars on forms of discrimination.” Student 6.

“This is not the first seminar I ever participated in about racism but it definitely is the first ever seminar in which I was asked to reflect about my experiences with racism in my privileged position and I admit that I was confused at first as to what I do have to contribute.” Student 10.

“The curriculum is slowly changing at the moment, but none of these lectures/seminars are mandatory. It is absurd that you can for example become a judge in migration law but might have never heard anything, at least in depth, about (anti)racism.” Student 6.

In their essays, several students commented on the broader institutional context of studying racism in Germany. The rarity of Racism Critique (Rassismuskritik) in German higher education institutions was criticized. One student (Student 6) critiqued the absence of anti-racism education in other disciplines of their study programs, such as law. Students highlighted how racism is often treated as a niche topic rather than an essential transdisciplinary knowledge-framework across fields. Another student noted that even within disciplines like sociology, race and racism are frequently addressed in a detached, theoretical manner, avoiding discussions of lived experience and the real-life impacts of structural power (Student 11). Some students expressed frustrations about the political resistance to structural change, with one student (Student 5) discussing the case of Hamburg's “Decolonizing Hamburg” project, where efforts to expose the city's colonial past met institutional pushback. Other students linked these forms of institutional resistance to a broader resistance in Germany to acknowledge race as a structural issue, citing the reluctance to collect race-based data (as a legacy of Third Reich eugenic racialization policies) as one example (Student 11). Some students called for a more explicit integration of anti-racist frameworks into university curricula, rather than relegating them to elective or temporary courses (Student 12).

The students' reflections highlight the strengths and limitations of studying race and racism critique in a digital, English-language seminar. In their estimating, some of the most pertinent tradeoffs for their learning included language accessibility vs. contextual relevance: English helped students engage with a broader discourse, but translating concepts into the German geopolitical context posed challenges. More explicit comparative discussions could help bridge this gap. Digital learning was experienced as both a safe and an isolating space. The Zoom platform enabled certain (self-) protective mechanisms but also reduced informal interactions and posed technical barriers. A hybrid or blended model could address this. For many students the course prompted reflection on whiteness and the unequal emotional labor of BIPoC in anti-racism classroom settings, but clearer guidelines for engagement and accountability could further support equity and accountability learning spaces. More structured facilitation could help redistribute the burden of discussing race and racism. Further reflection is required on the institutional challenges beyond the classroom space. Students recognized the limits of racism critique in German academia and society. Integrating more action-oriented components, such as partnerships with activist groups or policy discussions, could strengthen their engagement. Students' insights reveal how structural silences shape anti-racist learning, positioning them between institutional constraints and personal commitment.

### 5.1 Students' emotional and ethical reflections while discussing racism

“I'm white and was raised in a racist society, so it feels like a duty to me to educate myself and others in my surroundings about those racist norms and structures we live in, profiting from and even reproduce not even recognizing we're doing it. I feel like that there are many people who refuse to deal with this issue because they have to admit that they directly or indirectly benefit from racism and actually have to do something to change this status quo.” Student 5

“I find that as a white person living in a racist society, it is my obligation to educate myself on racism and antiracism. No matter how long I have been studying the subject, there is always something to (un)learn.” Student 6.

“The possibility to switch off the camera or to easily leave the room as a racialized or migrantized person could be a good possibility of protection in a potentially unsafe situation, which is to be in a conversation with white people about racism. For a privileged white person it can be a possibility to hide their own feelings of shame, guilt or just tiredness. I'm not sure if a white person should be able to switch off the camera in this setting.” Student 2.

Students reflected on their own racialized positionality in studying racism. Specifically, white students reflected on their emotional discomfort and also on accountability. Some white students viewed educating themselves on racism as a civic duty (Student 5, Student 6). A few students also acknowledged that discomfort prevents many privileged participants from engaging deeply with racialization and critique of racism (Student 5). One participant noted that white students may use the digital platform's features (e.g., turning off the camera) to avoid confronting feelings of guilt or complicity (Student 2). Another white student emphasized the importance of accepting some level of discomfort as a necessary part of unlearning racism, and at the same time recognized that many privileged students lack the tools to navigate discomfort productively (Student 10). Several BIPoC students who are negatively impacted by racism highlighted the unequal emotional labor apportioned to those marginalized by racist norms, structures and practices. While not explicitly stated, some reflections suggested an awareness that racialized students might have different stakes in the discussion around accountability and self-reflection. The idea of using the digital platform as a protective measure (Student 2, Student 4) hints at underlying concerns about being put on the spot or burdened with explaining racism to white peers. One student noted that the expectation for racialized students to provide personal testimonies about racism was emotionally exhausting and often led to tokenization (Student 11). Another suggested that more structured facilitation could help redistribute the burden of explaining race and racism (Student 10). This emotional work exemplifies trilemmatic positionality as students balance privilege, vulnerability, and institutional silence.

### 5.2 Language as a mediator and barrier in studying racism

“A lot more research seems to be done in English (and English-speaking countries), which is why the variety of resources is a lot higher in English. Therefore, it makes a lot of sense to have seminars on racialization in English.” Student 7.

Several students reflected on the complex positionality they inhabited while studying racism in Germany through the medium of English—a position shaped by the simultaneous pressures of institutional expectations, transnational academic norms, and their own linguistic and cultural identities. This can be understood as a form of trilemmatic positionality, where students were navigating between the institutional reliance on English-language scholarship, the critical project of engaging with racism in a meaningful way, and the limitations or privileges tied to their own linguistic capacities.

Students highlighted the benefits of working in English, particularly in terms of access to terminology and theoretical frameworks that are more extensively developed in English-speaking contexts. Several noted that English offered a richer vocabulary for engaging with concepts from Critical Race Theory and intersectionality (Student 1, Student 3, Student 7). Others emphasized how English enabled participation in broader, transnational discourses that would otherwise be less accessible within the German academic context (Student 8). Yet these advantages were also accompanied by tensions. The difficulty of translating key terms, such as “People of Color,” into German was noted as a source of conceptual and cultural friction, with students expressing concern that such translations could lead to misunderstandings or the erasure of local specificity (Student 1, Student 3).

This tension illustrates the trilemma at play: while English opened doors to global scholarly engagement, the lack of equivalent terminology in German made it challenging to localize and contextualize these frameworks within the unique post-unification racial landscape of Germany (Student 7, Student 9). Some students recognized that relying on English-language texts supported their engagement with global discourses, but also risked obscuring the specific histories, structures, and vocabularies of racism in Germany. Students shared awareness that racism is a global phenomenon with nation-specific forms, and some worried that English might flatten these distinctions, masking the particularities of the German race project (Student 1, Student 7). Conversely, others viewed English as a valuable tool for making German discussions legible to an international audience (Student 3, Student 8).

This trilemmatic positioning extended into questions of knowledge hierarchies and inclusion. Students critically reflected on how the dominance of English in academia reproduces power asymmetries, privileging Anglo-American frameworks and marginalizing locally grounded knowledge (Student 9). The expectation of English proficiency was also problematized. While some students recognized the practical necessity of English in academic contexts, they also noted that this expectation could be exclusionary. For students less fluent in English, participating fully in course discussions posed a challenge (Student 3), and one student pointed out that English fluency often served as an unspoken marker of academic legitimacy, reinforcing class-based inequalities (Student 9). Language itself became a site of negotiation for power, belonging, and epistemic authority.

### 5.3 Trilemmatic positionality and the digital learning format

The availability of recorded sessions (as lecturers of this course, we recorded only our own presentations) was particularly appreciated by those who faced internet or health-related disruptions, ensuring accessibility and sustained engagement. The student feedback highlights how digital platforms can both facilitate and complicate learning about power and exclusion. As technofeminist scholars have noted, technology is never neutral: it is shaped by and embedded within social hierarchies of race, gender, and class (Wajcman, 2004; Shivers-McNair et al., 2019). For some students, the online environment offered a measure of emotional safety—such as the option to switch off the camera or choose anonymized names during class discussions—particularly when engaging with emotionally taxing themes. For others, the digital platform amplified vulnerabilities, including a sense of invisibility or disconnection. These diverging experiences reinforce the need for reflexive digital pedagogies that attend to the differentiated ways in which power circulates on digital platforms.

As Benjamin (2019) warns in her analysis of the “New Jim Code,” we must be vigilant not to reproduce racial hierarchies through our use of technology. In our course, this meant recognizing how access, visibility, and participation were all mediated by structural inequalities. At the same time, we viewed digital tools as potentially transformational in nature, allowing us to co-create a learning environment that challenged normative knowledge hierarchies and fostered student agency. The feedback mechanisms we developed not only provided valuable insights into students' experiences, but also underscored the transformative potential of the course. By centering self-reflexivity and positionality, the seminar created a space where students could critically engage with both historical processes of racialization and their own situatedness within them.

“Zoom calls brings every participant on the same level, so it is not visible that there might be hierarchy in the zoom call—between teachers and students and also among students. Maybe because every participant has less options to dominate the class except through speaking, it has the potential to be more democratic.” Student 2.

“Students can decide whether they want to show their faces and/ or real names. It gives people the opportunity to stay silent and not participate or even leave, which is also a possibility in in- class environments but the hurdles are bigger.” Student 4.

“Going further, zoom gives the lecturers more control of the space, in the most extreme case, meetings can be ended with one click or participants can be removed from the room.” Student 4.

The use of Zoom as a learning environment emerged as a central theme in student reflections and can be analyzed through the lens of trilemmatic positionality. Students navigated the complex intersection of institutional constraints (e.g., digital infrastructures, language norms), pedagogical aims (e.g., anti-racist, inclusive teaching), and their own positional locations shaped by race, class, and access to resources.

Three key dimensions of this trilemma were reflected in students' experiences. The first centered around the idea of Zoom as a potentially “safer” space for students affected by racism or intersectional discrimination. Several students described how the online format offered more self-determined modes of participation, particularly for those from racialized or marginalized backgrounds. Features such as turning off the camera, modifying one's displayed name or pronouns, or leaving the session quietly were perceived as protective and empowering (Student 2, Student 4). This reflects a tension inherent in trilemmatic positionality: while institutional tools (like Zoom) can enable safer engagement with sensitive topics, they also carry the risk of facilitating avoidance. One student noted that the same mechanisms that allowed for self-protection could also enable disengagement or a withdrawal from accountability in moments of discomfort (Student 10). These simultaneous possibilities illustrate the fine balance required to create emotionally safe spaces while maintaining pedagogical rigor and responsibility.

The second dimension related to hierarchies and (the illusion of) democratization in digital learning spaces. Some students felt that Zoom minimized visible markers of social status, such as clothing or physical presence, which could help flatten classroom hierarchies (Student 2). Others, however, pointed out that the loss of body language and informal interaction made it harder to build trust and connection, limiting the emotional depth of classroom relationships (Student 6). Importantly, one student also emphasized that while visual hierarchies may be reduced, deeper power structures—including geopolitical asymmetries and linguistic dominance—remained intact. For instance, the predominance of English-speaking participants continued to shape classroom dynamics, despite the apparent equalizing effects of the digital platform (Student 9). Here, the trilemma emerges in students' efforts to participate meaningfully in a transnational, anti-racist learning space while navigating persistent inequalities within and beyond the virtual classroom.

The third category focused on technical and structural barriers, which further illustrate the conflicting pressures of trilemmatic positionality. While for some Zoom made the classroom more accessible, for others it posed significant challenges due to unstable internet connections, a lack of quiet learning environments, or the emotional exhaustion of screen-based engagement (Student 5, Student 6). The absence of informal, embodied interactions, such as hallway conversations before and after class, was also perceived as a loss (Student 6). One student noted that digital learning, though framed as more flexible, often reproduced existing inequalities: those with access to better technology and more stable living conditions were better positioned to benefit from the course (Student 11). Digital learning environments both reproduce and challenge racial hierarchies, revealing technology's ambivalent role in anti-racist education.

## 6 Conclusions and future research

Our reflections on teaching an online seminar in English at a German university reveal how anti-racist pedagogy unfolds within institutional, linguistic, and affective constraints. Using the framework of trilemmatic positionality, we analyzed how both educators and students navigate the tensions between institutional resistance, anti-racist commitments, and individual positionalities shaped by language, race, and access. This framework allows us to connect micro-level learning processes to broader structural dynamics in German higher education.

Three dimensions of trilemmatic positionality became central in our analysis:

First, institutional silences around race and racism persist in the German university context, shaping the conditions under which anti-racist learning takes place. When we talk about race and racism, West Germany's perspectives take front stage, largely ignoring GDR's history in shaping race and racism. Students' reflections revealed how racism remains marginal in curricula and is often treated as a specialized or elective topic rather than as a central component of social science education. This structural absence reproduces the very exclusions that anti-racist pedagogy seeks to challenge. Second, emotional and ethical labor emerged as a key dimension of anti-racist learning. Students occupying privileged positions described discomfort, guilt, or defensiveness, while racialized and migrantized students articulated the exhaustion of constantly having to explain racism. This imbalance highlights how institutional settings often leave the emotional responsibility for anti-racist engagement unequally distributed. Third, language and technology mediated learning in significant ways. Teaching in English allowed us to draw on global critical race theory, yet it also created a tension between global frameworks and the specific historical context of Germany. Similarly, the digital format opened new spaces for participation and safety but also risked isolation and disengagement, as students reported. Students' reflections show that both language and technology function as sites where power and access are negotiated in teaching. The course's online format also illuminated how digital environments are never neutral. Following Ruha Benjamin (2019) and Judy Wajcman (2004), we view digital spaces as politically and affectively charged: they can reproduce existing hierarchies but also enable new forms of agency and solidarity.

Building on these insights, future courses should create stronger links between academic study and activism. Engaging students in research on institutional diversity policies, or in collaborations with activist and community organizations, can help translate critical reflection into collective action. To sustain these efforts, universities must recognize anti-racist pedagogy as a legitimate field of knowledge production and provide institutional support for it.

## Data Availability

The raw data supporting the conclusions of this article will be made available by the authors, without undue reservation.

## Footnotes

1 The baseball bat years, a specific period in German history following reunification, were characterized by right-wing extremist violence and neo-Nazi activity, particularly in East Germany.

2 The reviewer of this paper asked us to clarify this concept in relation to Alyosxa Tudor's concept “migratization” (Tudor, 2018). Our term “migrantization” and Tudor's “migratization” are similar concepts. According to Tudor, migratization refers to the social process by which individuals or groups are ascribed the status of “migrant,” regardless of their actual migration history. Tudor's work explains how migratization helps analyze the power dynamics involved in labeling people as migrants. Tudor's research is considered foundational because they have theorized and coined the terms migratization and migratism, drawing parallels to concepts like ‘racialization' and ‘racism.' We have tremendous respect for Tudor's work. In our conceptualization, migrantization also points to the process by which racialized citizens are labeled as non-belonging to their nation-states, just as any kind of “foreigners” are in Germany. For example, in our classes, Black German students describe everyday encounters with white Germans who speak to them in English on the street, assuming that they are “foreigners” and do not speak German as their native language. Our emphasis in discussing migrantization is that people of color are rendered as non-belonging because whiteness is publicly conceived as the normative standard of citizenship and belonging in Germany (for further reading on our previous usage of this term, please see Yurdakul, 2009 and Yurdakul and Altay, 2023).

3 A total of 36 students signed up for our course. A very large part of the group were students with a trinational biography or migrantized students, who reported being relieved to be in a course with English as a language of instruction.

4 The student-curated course on orientalism was specifically important for them to discuss the ongoing effects of Israeli-Palestinian conflict in German society.
